# Assessing the Effects of Electroconvulsive Therapy on Cortical Excitability by Means of Transcranial Magnetic Stimulation and Electroencephalography

**DOI:** 10.1007/s10548-012-0256-8

**Published:** 2012-10-09

**Authors:** Silvia Casarotto, Paola Canali, Mario Rosanova, Andrea Pigorini, Matteo Fecchio, Maurizio Mariotti, Adelio Lucca, Cristina Colombo, Francesco Benedetti, Marcello Massimini

**Affiliations:** 1 Department of Biomedical and Clinical Sciences “L. Sacco”, Università degli Studi di Milano, Via G.B. Grassi, 74, 20157 Milan, Italy; 2 Department of Clinical Neurosciences, Scientific Institute and University Vita-Salute San Raffaele, Milan, Italy; 3 C.E.R.M.A.C. (Centro di Eccellenza Risonanza Magnetica ad Alto Campo), University Vita-Salute San Raffaele, Milan, Italy

**Keywords:** Cortical excitability, ECT, TMS/EEG, Neuromodulation, Major depression

## Abstract

Electroconvulsive therapy (ECT) has significant short-term antidepressant effects on drug-resistant patients with severe major depression. Animal studies have demonstrated that electroconvulsive seizures produce potentiation-like synaptic remodeling in both sub-cortical and frontal cortical circuits. However, the electrophysiological effects of ECT in the human brain are not known. In this work, we evaluated whether ECT induces a measurable change in the excitability of frontal cortical circuits in humans. Electroencephalographic (EEG) potentials evoked by transcranial magnetic stimulation (TMS) were collected before and after a course of ECT in eight patients with severe major depression. Cortical excitability was measured from the early and local EEG response to TMS. Clinical assessment confirmed the beneficial effects of ECT on depressive symptoms at the group level. TMS/EEG measurements revealed a clear-cut increase of frontal cortical excitability after ECT as compared to baseline, that was significant in each and every patient. The present findings corroborate in humans the idea that ECT may produce synaptic potentiation, as previously observed in animal studies. Moreover, results suggest that TMS/EEG may be employed in depressed patients to monitor longitudinally the electrophysiological effects of different therapeutic neuromodulators, e.g. ECT, repetitive TMS, and sleep deprivation. To the extent that depression involves an alteration of frontal cortical excitability, these measurements may be used to guide and evaluate treatment progression over time at the single-patient level.

**Electronic supplementary material:**

The online version of this article (doi:10.1007/s10548-012-0256-8) contains supplementary material, which is available to authorized users.

## Introduction

Electroconvulsive therapy (ECT) is generally recommended to drug-resistant patients suffering from severe major depression, because of its significant short-term antidepressant effects (Uk ECT Review Group 2003). The mechanism of action of ECT still needs to be fully characterized, although several hypotheses have been conceived, involving neurotransmitter systems (Yatham et al. 2010), endocrinological pathways (Kunugi et al. 2006), and neurogenesis (Nibuya et al. 1995; Madsen et al. 2000).

In animals, electroconvulsive seizures (ECS) have several effects on synaptic plasticity, gene transcription, and cell proliferation. ECS induces potentiation-like long-lasting synaptic changes (Stewart et al. 1994), increases the expression of neurotrophins, especially brain-derived neurotrophic factor (BDNF), and thus the activation of their receptor tyrosine kinase B (TrkB) (Nibuya et al. 1995; Altar et al. 2004) and stimulates the proliferation of neuronal and glial cells in the frontal cortex and hippocampus (Malberg et al. 2000; Madsen et al. 2005; Perera et al. 2007) of rats and nonhuman primates. BDNF/TrkB receptors promote neuronal function, growth and regeneration (Mamounas et al. 1995) and are mainly located at glutamatergic synapses, where they trigger synaptic potentiation and serve as activity-dependent modulators of synaptic plasticity (Mattson 2008; Minichiello 2009). Glial cells are also involved in the glutamatergic neurotransmitter system, by providing energy to neurons and releasing neurotrophic factors (Ben Achour and Pascual 2010).

On the other hand, clinical studies have associated stress and depression with atrophy and loss of neurons and glia, especially in the prefrontal cortex and hippocampus (Salvadore et al. 2011; Duman and Voleti 2012). An abnormally reduced expression of BDNF/TrkB has been observed in the frontal cortex and hippocampus of suicide patients (Dwivedi et al. 2003). Moreover, depressive symptoms have been correlated with dysfunction of the prefrontal cortex (George et al. 1994), particularly concerning its relationship with limbic structures implicated in the regulation of mood and anxiety (Alexander et al. 1986; Drevets 2000; Suwa et al. 2012).

The existence of a causal relationship among depression, plasticity and neurotrophins proposed by previous literature (Manji et al. 2001; Zarate et al. 2003) has encouraged to explore the modulation of motor cortex excitability induced by ECT in humans (Sommer et al. 2002; Chistyakov et al. 2005; Bajbouj et al. 2005, 2006). The results of these studies are not conclusive, since cortical excitability was found to be either decreased (Sommer et al. 2002; Bajbouj et al. 2005, 2006) or increased (Chistyakov et al. 2005) after treatment as compared to baseline. However, two of these studies (Sommer et al. 2002; Bajbouj et al. 2005) referred to single cases and all of them used the peripheral muscular responses to transcranial magnetic stimulation (TMS) of the primary motor cortex to estimate cortical excitability.

Today, a complementary, direct measure of cortical excitability in humans can be obtained non-invasively by means of transcranial magnetic stimulation combined with electroencephalography (TMS/EEG) (Ilmoniemi et al. 1997; Komssi and Kähkönen 2006; Ziemann 2011). This approach allows to directly perturb cortical regions and to record the immediate electrophysiological responses of the stimulated neurons. Most important, TMS-evoked potentials (TEPs) are characterized by high test–retest reproducibility, provided that stimulation parameters (site, intensity and angle) are accurately controlled across subsequent sessions by means of a magnetic resonance (MR)-guided navigation system (Casarotto et al. 2010). Hence, TMS/EEG has been successfully applied to detect, at the group level, cortical excitability changes induced by repetitive TMS (rTMS) in healthy individuals (Esser et al. 2006; Veniero et al. 2010). In these cases, the local synaptic potentiation deliberately induced in the motor cortex by high-frequency rTMS was measured as an increase of the amplitude of early TMS-evoked EEG responses between 0 and 80 ms (Esser et al. 2006; Veniero et al. 2010). Similarly, a recent TMS/EEG study (Huber et al. 2012) has allowed to detect a physiological increase of cortical excitability with time awake, that became significant at the single-subject level after one night of sleep deprivation.

In this work, we employ TMS/EEG to study the electrophysiological changes induced by ECT in human cortical circuits. Thus, we measured the changes in the immediate EEG response to TMS of the frontal cortex before and after a course of ECT. The aim of this study was to contribute to the understanding of the electrophysiological mechanisms of ECT in the human brain and to evaluate novel tools for monitoring and guiding neuromodulation protocols for the treatment of depression.

## Materials and Methods

The experimental protocol involved two TMS/EEG recording sessions for each patient, that were performed, respectively, the day before the first administration of ECT (pre-ECT) and the day after the last administration of ECT (post-ECT), early in the afternoon at about 3:00 pm. The same stimulation parameters were applied to each patient in both sessions.

### Participants

Eight inpatients suffering from Major Depressive Disorder, as diagnosed according to the DSM-IV criteria, participated in this study (Table 1). Physical examination, laboratory tests and electrocardiograms were performed at admission. All patients were referred to ECT because they were drug-resistant, i.e. showed a lack of improvement to at least two different treatments with antidepressants, at adequate dosage and duration (>6 weeks) (Fava 2003). No patient had received ECT within 6 months prior to study enrolment. Additional diagnosis on axis I besides major depression, mental retardation on axis II, history of drug/alcohol abuse or dependency, and major medical/neurological disorders were regarded as exclusion criteria. In addition, patients with medical history of seizures, convulsions, loss of consciousness and traumatic brain injury, carriers of intracranial metallic objects and/or of cardiac pace-makers were excluded to prevent potential adverse effects of TMS. Severity of the current major depression episode was rated on the 21-item Hamilton Depression Rating Scale (HDRS) (Hamilton 1960), administered by a qualified psychiatrist. HDRS was assessed at baseline, after each ECT session, and at the end of the whole treatment. All patients were properly acquainted with the experimental procedures (Mini-Mental State Examination was always above 26) before signing a written informed consent to participation. Moreover, we explicitly reassured them about the possibility of interrupting the experiment upon any unpleasant feeling. The study was approved by the Local Ethical Committee.

**Table 1:** Demographic characteristics

Patient	Gender	Age (years)	Age of onset of depression	Family history	*n*° previous episodes	Duration current episode (months)	Concurrent drug treatment
1	F	54	50	No	2	16	TCA,NL
2	M	42	28	No	2	24	TCA
3	F	60	57	No	1	24	TCA
4	M	53	31	Yes	2	36	TCA
5	F	44	15	Yes	3	6	SNRI
6	F	51	35	Yes	2	24	TCA
7	F	61	41	Yes	7	7	SNRI
8	F	51	39	Yes	3	12	SSRI

*F* female, *M* male, *SNRI* serotonin norepinephrine reuptake inhibitors, *TCA* tricyclics, *NL* neuroleptics, *SSRI* selective serotonin reuptake inhibitors

### ECT Delivery

ECT was administered twice a week with bilateral electrode placement (spECTrum 5000Q^®^, MECTA, Tualatin, OR, USA). Two circular flat electrodes (diameter: 5.08 cm) were positioned on both sides of the head with midpoints approximately 2.54 cm above the center of a line connecting the tragus and the external canthus. Current was delivered through trains (duration: 1.39 ± 0.13 s) of rectangular, constant-current pulses (amplitude: 800 mA) with alternating polarity (pulse width: 1.41 ± 0.12 ms; pulse pairs per second: 57.36 ± 7.36 Hz). The dose titration method for eliciting adequate seizures and other technical parameters were established according to (American Psychiatric Association. Task Force on Electroconvulsive Therapy. 2001). Brief general anesthesia was induced with thiopental (i.v. 5 mg/kg) and succinylcholine (i.v. 1 mg/kg) for relaxation. In addition, pre-medication with atropine (i.m. 0.5 mg/kg) was used to reduce the risk of vagal immediate bradyarrhythmia or asystole. Each patient was administered a variable number of ECT sessions, according to the individual clinical needs evaluated by experienced clinicians (Table 2). Each patient was carefully evaluated by qualified psychiatrists throughout the whole ECT protocol, in order to monitor the therapeutic and cognitive effects of treatment. In particular, ECT was administered until remission of depressive symptoms (at least 50 % reduction of HDRS score as compared to pre-ECT assessment) or onset of unwanted side effects pertaining cognitive functions.

**Table 2:** Clinical and neurophysiological effects of ECT

Patient	*n*° ECT sessions	HDRS			IRA (μV^2^)				IRS (μV/ms)		
		Pre-ECT	Post-ECT	*p*	Pre-ECT	Post-ECT	*p*		Pre-ECT	Post-ECT	*p*
1	8	38	18		47	58	.034		0.57	0.80	.006
2	7	27	13		74	82	.014		0.76	1.07	.000
3	3	22	3		57	75	.020		0.64	0.67	.771
4	8	20	12		9	18	.026		0.04	0.46	.004
5	7	25	16		132	196	.002		0.21	0.52	.000
6	9	19	12		14	23	.010		0.09	0.14	.268
7	5	25	2		9	21	.002		0.15	0.31	.000
8	3	19	3		24	33	.024		0.36	0.37	.717
Mean ± SE	6.3 ± 0.82	24.4 ± 2.22	9.9 ± 2.23	.025	45.7 ± 14.94	63.2 ± 20.90	.025		0.35 ± 0.1	0.54 ± 0.11	.025

*SE* standard error, *ECT* electroconvulsive therapy, *HDRS* Hamilton Depression Rating Scale, *IRA* immediate response area, *IRS* immediate response slope

### TMS/EEG Recording

TMS was delivered with a Focal Bipulse 8-Coil (mean/outer winding diameter ca. 50/70 mm, biphasic pulse shape, pulse length ca. 280 μs, focal area of the stimulation hot spot 0.68 cm^2^; Eximia TMS Stimulator, Nexstim Ltd., Helsinki, Finland). Stimulation parameters were controlled by means of a Navigated Brain Stimulation (NBS) system (Nexstim Ltd., Helsinki, Finland), that employs a 3D infrared Tracking Position Sensor Unit (Polaris, Northern Digital Inc., Waterloo, Canada) and integrates T1-weighted structural MR images recorded from all patients (3.0 tesla scanner, Intera, Philips Medical Systems, Best, The Netherlands; 0.9 × 0.9 × 0.8 mm spatial resolution). This equipment maps in real time the positions of TMS coil and subject’s head within the reference space of individual MR images by aligning the fiducials selected on structural images with the corresponding digitized scalp landmarks (nasion, left and right tragus). The NBS system (Ruohonen and Ilmoniemi 1999; Ruohonen and Karhu 2010) allowed (i) to precisely stimulate a cortical target selected a priori on individual MR images, (ii) to estimate the electric field (EF) induced on the cortical surface by TMS pulses, and (iii) to accurately repeat the same stimulation parameters between sessions. The induced EF clearly depends on stimulation intensity, expressed as percentage of the maximal stimulator output, on the relative position between subject’s head and TMS coil, and on the geometrical and physical properties of head tissues between the stimulator and the cortical surface. The distribution and intensity (expressed in V/m) of the intracranial induced EF was estimated on-line using a locally best-fitting spherical model of the subjects’ head and brain and taking into account the exact shape, 3D position and orientation of the TMS coil. Precise control over stimulation coordinates across sessions was obtained by means of a virtual aiming device, that displayed in real time on a screen any deviation from the desired target greater than 3 mm and therefore allowed manual adjustments of the stimulator position.

The middle-caudal portion of the superior frontal gyrus close to the midline (near the boundary between Brodmann areas 6 and 8) was selected as target area. Indeed, the prefrontal cortex is maximally involved by current flow during bilateral ECT (Boylan et al. 2001; Lee et al. 2012) and its metabolic activity is significantly modulated by treatment (Nobler et al. 2000; Suwa et al. 2012). Moreover, this targeting ensured maximal distance from cranial muscles, which are mainly distributed over the lateral surface of the scalp and whose unwanted activation by TMS would affect EEG recordings. TMS pulses were delivered to the target area nearby the mid-sagittal plane: however, since TMS is not suited to properly stimulate along the longitudinal fissure (Thielscher et al. 2011), the coil was slightly moved either towards the left or the right side of the head in order to better deliver TMS pulses on the convexity of the middle-caudal portion of the superior frontal gyrus, with the current perpendicular to its main axis (Supplementary Fig. 1S). Patients were randomly divided into two groups of equal size for being predominantly stimulated either on the left (four patients) or right (four patients) hemisphere. This protocol was applied to obtain a comparable amount of data from stimulation of both hemispheres in a small group of patients, in the lack of a priori assumptions about laterality of the effects of bilateral ECT. Moreover, this approach allowed to shorten the duration of TMS/EEG sessions, thus limiting any additional stress to patients. Two patients were particularly collaborative and therefore agreed to being stimulated on both hemispheres (Supplementary Fig. 1S). Excitation threshold has been reported to change with cortical location (Stewart et al. 2001; Boroojerdi et al. 2002; Gerwig et al. 2003); moreover, we specifically delivered TMS pulses over the prefrontal cortex. Therefore, we set stimulation intensity relying on the actual intracranial induced EF estimated by the NBS system rather than referring to the minimum stimulator output needed to produce a reliable electromyographic response (motor threshold; Rossi et al. 2009). In all patients, the maximum EF induced by TMS on the cortical surface was kept in the range 90–130 V/m by means of the NBS system (corresponding to 56–75 % of the maximal stimulator output). Previous works have shown that this stimulation intensity elicits EEG responses with good signal-to-noise ratio (Komssi et al. 2007; Rosanova et al. 2009; Casali et al. 2010). The location of the maximum EF induced by TMS on the cortical surface was labeled TMS hotspot and was always located within the target area. Inter-stimulus interval was randomly jittered between 1500 and 1800 ms (equivalent to ca. 0.56–0.67 Hz). This stimulation rate does not induce significant reorganization/plasticity processes that might possibly either affect EEG responses to TMS or interfere with the longitudinal measurements (Casarotto et al. 2010). During TMS stimulation, patients wore inserted earplugs continuously playing a masking noise that abolished the auditory potentials elicited by TMS-associated clicks (Massimini et al. 2005).

A 60-channel TMS-compatible EEG amplifier (Nexstim Ltd., Helsinki, Finland) was used to record artifact-free electrical brain responses to single TMS pulses (Virtanen et al. 1999). On average, 247 ± 11 (mean ± standard error) pulses were delivered in each session (range 199–332 pulses). Impedance at all electrodes was kept below 5 kΩ. EEG signals were band-pass filtered between 0.1 and 500 Hz and sampled at 1,450 Hz with 16 bit resolution. In order to monitor ocular movements and blinks, vertical electrooculogram was recorded with two extra sensors. The position of EEG electrodes on the scalp was digitized and provided to the NBS system for integration with individual MR images and for allowing a precise replacement of the EEG cap between sessions.

### Data Analysis

Data analysis was carried out using MATLAB^®^ (2006a, The MathWorks, Natick, MA, USA). Principal Component Analysis was applied to automatically reduce ocular artifacts (Casarotto et al. 2004). Single TEPs residually contaminated by muscular activity (absolute power of EEG channel F8 above 25 Hz > 0.9 μV2/Hz) (van de Velde et al. 1998) were automatically rejected. After averaging a minimum of 90 artifact-free trials (mean ± standard error across sessions: 127 ± 10 trials; range 90–189 trials), channels containing high-frequency muscular activity or large residual artifacts were excluded from further analysis. Bad channels (always fewer than ten) were usually contaminated by high-frequency muscular activity and were mostly located peripherally over fronto-temporal muscles. Signal quality of channels nearby the stimulated site was always acceptable. In order to apply data analysis to the same number of channels across sessions and patients, we have interpolated bad channels using “Matlab 4 griddata method” (Sandwell 1987). Artifact-free signals were band-pass filtered between 2 and 80 Hz, down-sampled to 725 Hz, and re-referenced to the common average reference.

**Fig. 1:**
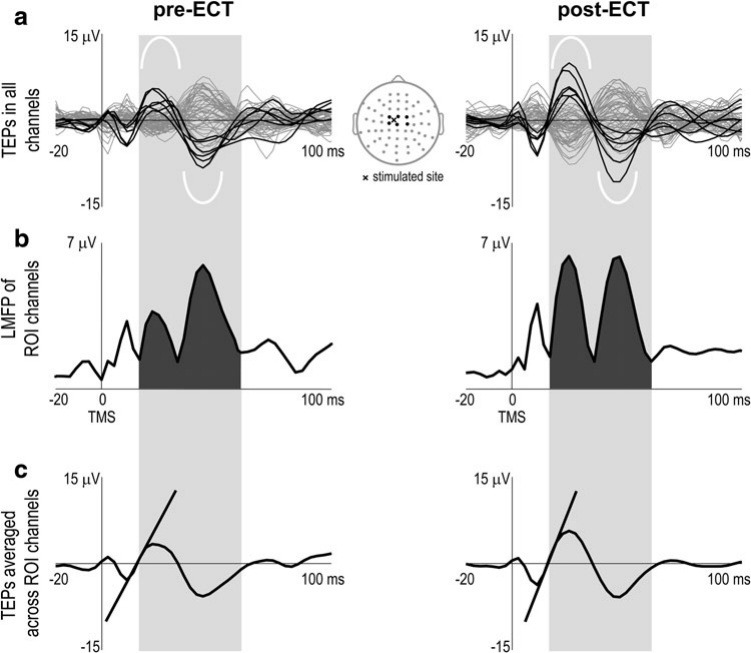
Computation of cortical excitability in patient 1. **a** Lateral plots represent the average TEPs superimposed in all EEG channels before (pre-) and after (post-) ECT. Central map depicts the electrodes arrangement (*black and gray dots*) on the scalp. *Black traces* correspond to ROI channels, located nearby the stimulated site (*black cross*) and containing a large, early TEP component, consisting in a positive wave (*white reversed U-shaped trace*) followed by a negative wave (*white U-shaped trace*). **b** LMFP of the ROI channels. Cortical excitability was measured by the subtended area (*dark gray shadow*) between the two local minima (*light gray shadow*) encompassing the early consecutive positive and negative waves triggered by TMS (IRA). **c** TEPs averaged across the ROI channels in the two conditions. Slanting lines highlight the slope of the rising side of the large positive wave early evoked by TMS (IRS)

We measured cortical excitability from the immediate EEG response to TMS. In all patients, TMS triggered a large, early TEP component, consisting in a positive wave (Fig. 1a, white reversed U-shaped trace) followed by a negative wave (Fig. 1a, white U-shaped trace) and with maximum amplitude in the electrode overlying the TMS target. We selected for each patient the six neighboring EEG channels (region-of-interest (ROI)) where this component had the largest amplitude (Fig. 1a, black traces) and we quantified two morphological features of this component, the immediate response area (IRA) and slope (IRS). IRA was obtained from the local mean field power (LMFP) of the TEPs across the ROI channels (computed as the square root of squared TEPs averaged across the ROI channels; Fig. 1b) (Lehmann and Skrandies 1980): in particular, we detected the two local minima encompassing the early consecutive positive and negative evoked waves (light gray shadow, Fig. 1), and then we calculated the integral of the LMFP within this interval (dark gray shadow, Fig. 1b). Since TEPs were highly reproducible across sessions, we used the same time interval in both pre- and post-ECT sessions. IRS was computed on single-trial TEPs averaged in space across the ROI channels: specifically, it was defined as the mean first derivative of the rising side of the positive wave (Vyazovskiy et al. 2008) of the early TMS-evoked component (Fig. 1c). Identification of the ROI channels, of the early TMS-evoked component, and of the local minima of the LMFP was performed for each patient separately with a computerized procedure and was then verified manually.

In order to study the effects of ECT at the group level, we applied a non-parametric paired sign test to compare the HDRS score, IRA, and IRS values between pre- and post-ECT sessions across patients. In addition, IRA and IRS values were statistically compared between sessions at the single-subject level. In the case of IRA, we applied the following non-parametric permutation-based statistical analysis: (i) under the null hypothesis of equivalence between pre- and post-ECT recordings, 1000 “mixed” TEPs were constructed by averaging single trials randomly selected from the two sessions; (ii) LMFP was computed from these surrogate TEPs and was used to estimate the empirical null distribution of IRA; (iii) cortical excitability in each patient was considered significantly affected by ECT with probability of false positives α when the actual IRA values laid beyond the α-th percentile tails of the null distribution. IRS was compared between sessions by non-parametric Wilcoxon rank sum test applied to single-trial slope values in each patient.

## Results

**Fig. 2:**
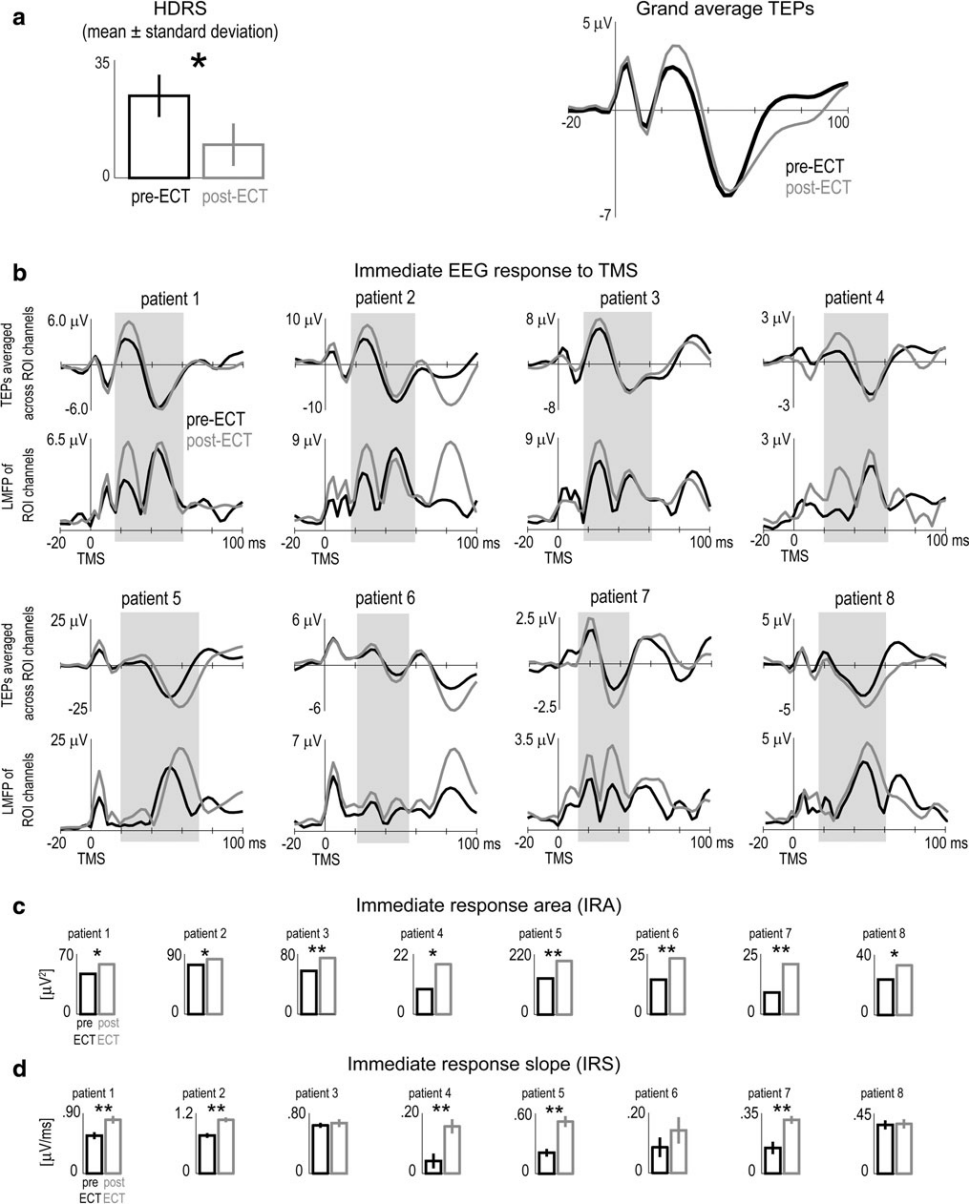
**a** (*Left*) At the group level, the HDRS assessed after (*gray bar*) ECT was significantly smaller (*p* < .025) than before treatment (*black bar*). (*Right*) Superimposition of grand average TEPs collected before (*black trace*) and after (*gray trace*) ECT. **b** Individual time courses of the TEPs averaged across ROI channels and of the LMFP of ROI channels before (*black traces*) and after (*gray traces*) ECT. **c** Single-subject comparisons (using permutation-based statistics) between cortical excitability, as measured by the IRA, before (*black bars*) and after (*gray bars*) ECT. **d** Single-subject comparisons (Wilcoxon rank sum test) between the IRS before (*black bars*) and after (*gray bars*) ECT. See *Materials and Methods* section for the individual selection of ROI channels and definition of IRA and IRS. *p < .05; **p < .005

At the group level, the clinical effect of ECT was a significant reduction of the HDRS score (*p* < .025) as compared to baseline values (Table 2, Fig. 2a left panel). TEPs displayed similar morphology across patients and were characterized by an early evoked component consisting of a positive wave between 13 ± 2 and 27 ± 5 ms followed by a negative wave peaking at about 47 ± 5 ms; the grand average TEP across patients (Fig. 2a right panel) shows that the amplitude of this component was clearly increased after ECT. IRA and IRS values were significantly increased at the group level after ECT (Table 2). Moreover, the increase of IRA after ECT as compared to baseline was significantly correlated with the corresponding increase of IRS (Pearson’s correlation = .86, R^2^ = .70, *p* < .006).

Statistical analysis further confirmed that the modulation of cortical excitability brought about by ECT was significant at the single-subject level (Fig. 2; Supplementary Fig. 2S). Fig. 2b displays the time course of individual TEPs and LMFP within the ROI channels. The morphology of the immediate EEG response to TMS was generally comparable among individuals and was highly reproducible between pre- and post-ECT sessions in the same patient. Most important, in all patients we observed an increase of the consecutive positive and negative waves early evoked by TMS and a corresponding increase of the LMFP after ECT. Non-parametric statistical comparison between pre- and post-ECT sessions showed that IRA was significantly increased in each and every patient (Table 2; Fig. 2c), while IRS increase was significant in all but 3 patients (Table 2; Fig. 2d; patients 3, 6, 8).

At the group level, the percentage reduction of HDRS by treatment was inversely correlated with the number of ECT sessions (Pearson’s correlation = −.89, R^2^ = .75, *p* < .004), indicating that mood improved more in patients with a faster response to ECT. A positive trend was observed between the percentage reduction of HDRS in post-ECT compared to pre-ECT session and the corresponding percentage increase of IRA, although correlation was not statistically significant (Pearson’s correlation = .53, R^2^ = .28, *p* < .18).

## Discussion

This study provides the first experimental evidence that ECT increases frontal cortex excitability in patients affected by severe major depression. This result was obtained by measuring the immediate (early and local) EEG response to a direct perturbation with navigated TMS.

Previous attempts to measure cortical excitability during a course of ECT were performed on the peripheral motor evoked potentials (electromyogram (EMG)) induced by TMS of the primary motor cortex (Sommer et al. 2002; Chistyakov et al. 2005; Bajbouj et al. 2005, 2006). While single-case studies (Sommer et al. 2002; Bajbouj et al. 2005) showed a reduction of cortical excitability after ECT (increase of intracortical inhibition, cortical silent period duration, and resting motor threshold), group results (Chistyakov et al. 2005) indicated an increase of cortical excitability as reflected by reduced motor threshold and intracortical inhibition. In the present study, we stimulated the frontal cortex, which is mostly involved by ECT effects (Boylan et al. 2001; Lee et al. 2012), and we analyzed the local EEG responses immediately triggered in this area by TMS. Notably, the TMS/EEG approach proposed here provides a direct measure of cortical excitability that is complementary to the TMS/EMG approach, because it by-passes spinal motorneurons and because it is applicable outside the motor cortex.

Developing TMS/EEG as a clinical tool for monitoring plastic/excitability changes in cortical circuits requires an adequate protocol for recording and analyzing data. The TMS/EEG apparatus used in this work was equipped with a neuronavigation system and took into account individual anatomical variability (Ruohonen and Karhu 2010). Previous works have shown that TMS-evoked responses recorded several days/weeks apart under the same experimental conditions are statistically identical (Lioumis et al. 2009; Casarotto et al. 2010): therefore, the significant TEP changes we observed in the same patient between pre- and post-ECT sessions cannot be ascribable to unknown experimental variables or measurement uncertainty, but are actually due to significant changes in neural responsiveness. The reliability of TEPs is strictly contingent on the use of neuronavigation, which ensures a reproducible targeting across sessions. Hence, MR-guided navigated stimulation may be an important requirement for the application of TMS/EEG as a clinical monitoring tool.

We found that the early response of frontal cortical neurons to a direct stimulation was significantly larger after a course of ECT treatment as compared to baseline. This result was observed in a rather limited population of patients; however, the effect was strong and consistent, since IRA increase was statistically significant in each and every patient. Whether this increase in cortical excitability reflects synaptic potentiation as suggested by animal models of ECT (Stewart et al. 1994; Nibuya et al. 1995; Altar et al. 2004) remains an open question. Indeed, the complex relationship between depression and neuroplasticity clearly involves multiple signaling cascades that regulate neuronal activity, including inhibitory neurotransmitters (Sackeim 2004), besides the glutamatergic system (Sanacora et al. 2008; Biedermann et al. 2011). Nonetheless, the possible involvement of neuroplasticity processes might be supported by the observation that also the slope of early TEPs was increased. Indeed, in the animal model, changes in slope of the first local field potential component elicited by electrical stimulation of cortical axons reflect changes in excitability related to the strengthening or weakening of cortical synapses. Accordingly, in vivo long-term potentiation-inducing procedures increase the local field potential slope (Bliss and Lomo 1973; Stewart et al. 1994), whereas long-term depression-inducing procedures reduce it (Kirkwood et al. 1993). Actually, IRS increase was significant in most, but not all, of the patients: however, it is challenging to properly record the local and immediate neuronal response to TMS, due to unwanted activation of cranial muscles nearby the stimulated site. Therefore, although we succeeded in recording good-quality early TEPs from most patients, IRA might be an alternative, more reliable measure of cortical excitability with respect to IRS in a clinical context. Nonetheless, finding a significant increase of IRS at the single-subject level in most patients further supports the possible interpretation of increased cortical excitability in terms of neuroplasticity processes.

Previous studies have related the neurobiological substrates of depression to an inter-hemispheric imbalance (Davidson and Irwin 1999; Maeda et al. 2000; Hecht 2010): indeed, emotions seem to be differently processed in the left and right hemisphere (Grimm et al. 2006) and cerebral blood flow and metabolism at rest have been observed to be abnormally reduced in the left prefrontal cortex and abnormally increased in the right prefrontal cortex in major depressed patients as compared to healthy subjects (Mayberg 2003). Moreover, therapeutic repetitive TMS is clinically delivered at a high-frequency rate over the left hemisphere (to increase cortical activity) and at a low-frequency rate over the right hemisphere (to suppress cortical activity) (Fitzgerald et al. 2003; Gershon et al. 2003). As a consequence, it is possible that the mechanism of action of any antidepressant treatment could be in principle affected by this asymmetry. The present study was not intended to specifically investigate the electrophysiological effects of ECT in the left and right hemisphere separately: rather, TMS pulses were delivered on the scalp nearby the mid-sagittal plane, slightly on the left side of the head in half of the patients and slightly on the right side in the other half (see *TMS/EEG recording* paragraph and Supplementary Fig. 1S). Basically, we stimulated mainly one hemisphere in a region close to the midline and we involved, to a lesser extent, also the contralateral hemisphere. The possibility that we actually obtained a direct stimulation of both hemispheres is supported by observing that the induced EF peaking at 90–130 V/m in the TMS target area actually activated above threshold a cortical area of several cm^2^, as estimated by the navigation system, and that the ROI channels with the largest early TMS-evoked EEG response were usually located bilaterally (Supplementary Fig. 1S). In two patients we were able to record EEG responses to stimulation of both hemispheres: results revealed a significant increase of both IRA and IRS in each patient bilaterally (Supplementary Fig. 2S). These observations confirm that ECT induces a comparable increase of cortical excitability on both sides of the brain, as could be expected by the bilateral electrode placement used in this therapeutic protocol. Clearly, most of the patients were prevalently stimulated on one hemisphere and therefore we could have missed the observation of a possible cortical excitability imbalance at baseline. This issue should be considered in future studies on larger sample size by comparing the immediate EEG response to TMS of the left and right hemisphere in the same patients.

The present results, showing increased frontal cortical excitability after ECT, fit with the observation that while stress reduces the expression of BDNF in the hippocampus and frontal cortex, antidepressants produce the opposite effect and promote neurons/glia survival and growth (Manji et al. 2001; Dwivedi et al. 2003; Salvadore et al. 2011; Duman and Voleti 2012). Accordingly, ECT increases neurotrophins, e.g. BDNF (Nibuya et al. 1995; Altar et al. 2004) and synaptic efficacy (Stewart et al. 1994), and BDNF/TrkB potentiate cortical excitability through interaction with glutamate and its receptors (Mamounas et al. 1995; Mattson 2008; Minichiello 2009). In humans, the glutamate/glutamine levels as measured by proton MR spectroscopy have been reported to be significantly smaller at baseline in depressed patients as compared to healthy subjects and to increase after successful ECT (Michael et al. 2003).

We observed a positive, yet not significant, trend between IRA increase and mood improvement as measured by HDRS. Although we do not aim to convey any prognostic information due to our limited sample size, we propose to further investigate the relationship between clinical and electrophysiological measurements of neuromodulatory treatments on larger populations, possibly accounting for confounding factors, e.g. gender, disease severity, or social influences.

A limitation of the present study consists in the lack of a control group, i.e. a comparable group of depressed patients treated with “sham” ECT, due to ethical reasons and to the limited number of available patients. Previous studies have necessarily applied a classical randomized placebo-controlled design to demonstrate the therapeutic efficacy of ECT (Freeman et al. 1978; West 1981). However, we took for granted the general antidepressant effects of ECT and we specifically focused on the electrophysiological modifications induced by this neuromodulatory treatment in frontal cortical circuits. Therefore, for our purposes it was unethical to preclude severely depressed patients at high risk from receiving a generally effective treatment and, at the same time, to submit them to a dose of anesthetic. Since we observed a significant increase of cortical excitability after ECT in each and every patient, despite the reduced sample size, we believe it is very unlikely that an unknown and uncontrollable variable other than ECT would have been able to significantly modulate TEPs consistently across patients. This study showed a clear-cut neurophysiological effect of ECT on cortical excitability, that could be detected statistically at the single-subject level: therefore, it suggests that a new tool based on TMS/EEG could be available to guide and monitor different antidepressant treatments. Clearly, ECT engenders a coarse effect on brain circuits. Thus, it would be interesting to employ TMS/EEG study the effects of more refined neuromodulatory treatments, e.g. repetitive TMS (O’Reardon et al. 2007; Padberg and George 2009; Slotema et al. 2010). rTMS is a non-convulsive mean to induce neuromodulation that does not require anesthesia, and has negligible side effects compared to ECT. In addition, the local activation of prefrontal cortical circuits by rTMS has been shown to indirectly affect subcortical regions involved in mood regulation (Li et al. 2004). Still, the antidepressant effects of rTMS are weaker as compared to ECT (Knapp et al. 2008) and can be unpredictable at the single-patient level (Hasey 2001; Eranti et al. 2007), possibly because of inadequate optimization of stimulation parameters, e.g. frequency, intensity, targeting (Hasey 2001; Lam et al. 2008). To the extent that an increase of frontal cortex excitability is the desired electrophysiological effect of antidepressant treatment, TMS/EEG measures may represent a reliable tool to quantify objectively and optimize therapeutic neuromodulation and may be used to identify effective stimulation parameters on an individual basis. For example, IRA can be calculated automatically from a small subset of electrodes nearby the TMS target and is able to detect significant effects at the single-subject level. Therefore, simpler experimental set-ups could be developed to study the effects of different treatments in larger cohorts of depressed patients. In future studies TMS/EEG set-ups may be used to evaluate the effects of other treatments, such as sleep deprivation, rTMS, and transcranial direct current stimulation.

## Electronic supplementary material

Below is the link to the electronic supplementary material.

Supplementary material 1 (TIFF 1365 kb)

Supplementary material 2 (TIFF 2133 kb)

Supplementary material 3 (DOCX 14 kb)
