# Supplementary figures and images for: Assessing the Effects of Electroconvulsive Therapy on Cortical Excitability by Means of Transcranial Magnetic Stimulation and Electroencephalography
Source: Brain Topogr. 2012 Oct 9;26(2):326–37. doi: 10.1007/s10548-012-0256-8 (PMC3587686; doi:10.1007/s10548-012-0256-8)

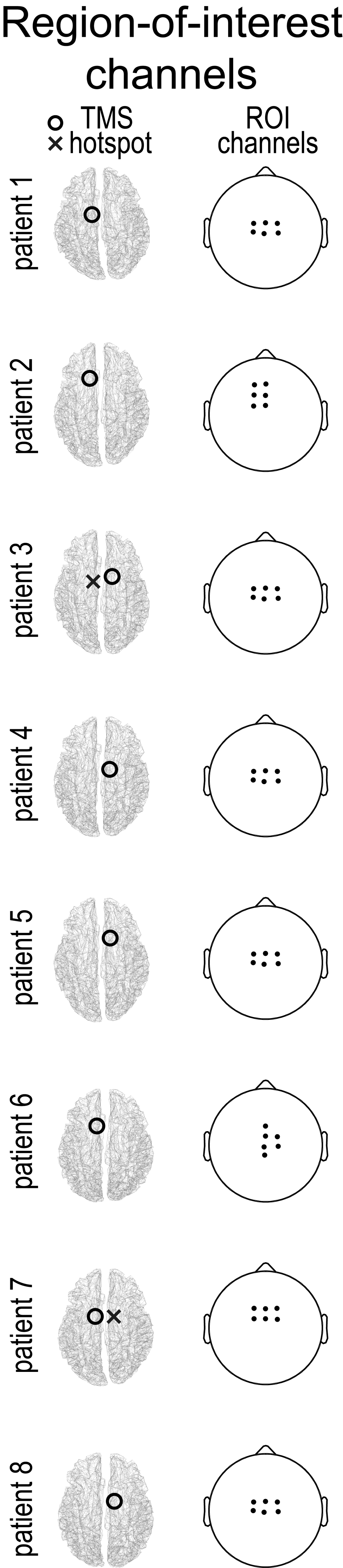

Supplement: Supplementary file 1 — Supplementary material 1 (TIFF 1365 kb) [file 10548_2012_256_MOESM1_ESM.tif]

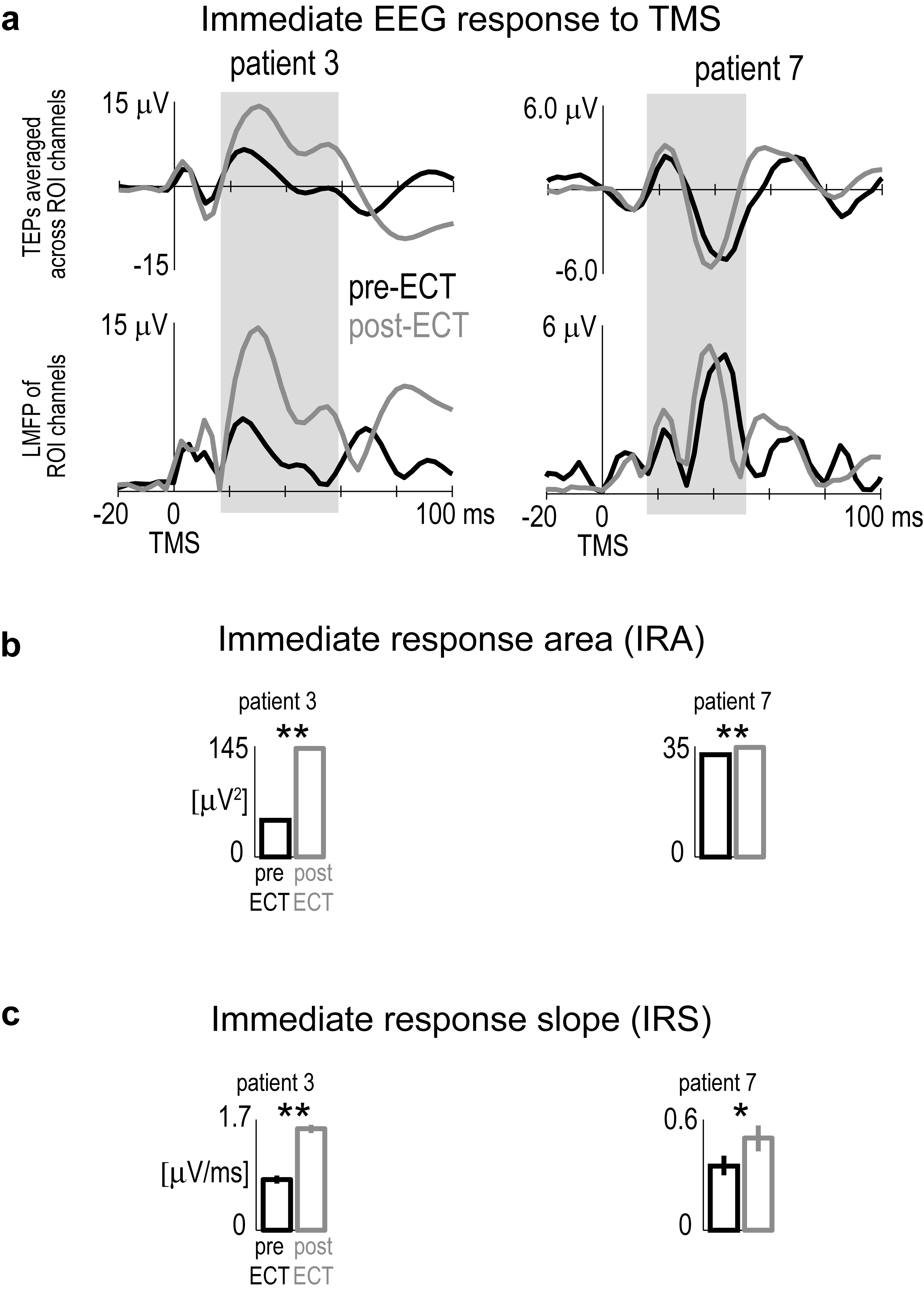

Supplement: Supplementary file 2 — Supplementary material 2 (TIFF 2133 kb) [file 10548_2012_256_MOESM2_ESM.tif]
