# Supplementary material for: Assessing the Effects of Electroconvulsive Therapy on Cortical Excitability by Means of Transcranial Magnetic Stimulation and Electroencephalography
Source: Brain Topogr. 2012 Oct 9;26(2):326–37. doi: 10.1007/s10548-012-0256-8 (PMC3587686; doi:10.1007/s10548-012-0256-8)
Supplement: Supplementary file 3 — Supplementary material 3 (DOCX 14 kb) [file 10548_2012_256_MOESM3_ESM.docx]

**Supplementary Figure legends**

**Fig. S1** (Left) Individual TMS hotspots (black circles), i.e. location of the maximum electric field induced by TMS on the cortical surface, and (right) region-of-interest (ROI) channels (black dots) reflecting the local electroencephalographic (EEG) response to TMS. In patients 3 and 7, it was possible to additionally record the EEG potentials evoked by TMS of the contralateral hemisphere (black crosses). In these session pairs, the immediate EEG response to TMS had largest amplitude in the same set of ROI channels.

**Fig. S2** Results of additional TMS/EEG sessions recorded from patients 3 and 7. **(a)** Individual time courses of the TEPs averaged across ROI (region-of-interest) channels and of the local mean field power (LMFP) of ROI channels before (black traces) and after (gray traces) ECT. **(b)** Single-subject comparisons (using permutation-based statistics) between cortical excitability, as measured by the immediate response area (IRA), before (black bars) and after (gray bars) ECT (patient 3: pre-ECT = 48 µV^2^, post-ECT = 142 µV^2^; patient 7: pre-ECT = 32 µV^2^, post-ECT = 35 µV^2^). **(c)** Single-subject comparisons (Wilcoxon rank sum test) between the immediate response slope (IRS) before (black bars) and after (gray bars) ECT (patient 3: pre-ECT = 0.78 ± 0.05 µV/ms, post-ECT = 1.56 ± 0.04 µV/ms; patient 7: pre-ECT = 0.35 ± 0.05 µV/ms, post-ECT = 0.48 ± 0.07 µV/ms). See *Materials and Methods* section for the individual selection of ROI channels and definition of IRA and IRS. * *p* < .05; ** *p* <.005
